# Antiproliferative, Cytotoxic, Antioxidant Activity and Polyphenols Contents in Leaves of Four *Staphylea* L. Species

**DOI:** 10.3390/molecules14093259

**Published:** 2009-08-28

**Authors:** Lubica Lacikova, Marianna Jancova, Jan Muselik, Irena Masterova, Daniel Grancai, Maria Fickova

**Affiliations:** 1 Department of Pharmacognosy and Botany, Faculty of Pharmacy, Comenius University, Odbojarov 10, 832 32 Bratislava, Slovakia; E-mail: lacikova@fpharm.uniba.sk (L.L.); 2 Department of Pharmaceutics, Faculty of Pharmacy, University of Veterinary and Pharmaceutical Sciences, Palackeho 1-3, 612 42 Brno, Czech Republic; E-mail: muselik@vfu.cz (J.M.); 3 Institute of Experimental Endocrinology SAS, Vlarska 3, 833 06 Bratislava, Slovakia

**Keywords:** *Staphylea*, antiproliferative, cytotoxic, antioxidant

## Abstract

*Staphylea* has been used for long time in Traditional Chinese Medicine (TCM) and by Native Americans in a number of therapeutical indications. The present study describes *in vitro* antiproliferative, cytotoxic properties (MTT and LDH test) and antioxidant activities (reduction of DPPH radical and peroxynitrite radical) of *Staphylea colchica* Stev. (SC), *S. elegans* Zab. (SC), *S. holocarpa* Hemsl. (SH) and *S. pinnata* L. (SP) leave water extracts. Time- (24 and 72 h) and dose- (1-150 μg/mL) dependent effects of the above extracts were tested at the mitochondrial (MTT test) and plasma membrane level (LDH leakage) in A431 human skin carcinoma cells. Screening of these properties has shown time and dose dependent increase of harmful effects, the highest activity was observed for the SE, while the less active was the SH extract. The ED_50 _values for the mitochondrial and membrane damage were nearly identical for the SE and very similar for SH extract. These findings indicate simultaneous injury of both cell compartments by SE and SH extracts. The highest antioxidant potential of SE species is accompanied by the highest content of flavones/flavonols and polyphenols. Only flavonoid contents are associated with antiproliferative effects and cell membrane injury, while antioxidant properties are the result of polyphenol content. The data clearly demonstrate that individual *Staphylea* L. species differ, not only in the amount of biologically active compounds, but also by the extent of harmful and beneficial effects.

## 1. Introduction

Bladdernut (*Staphylea* L., Staphyleaceae), belongs to the well-known group of ornamental shrubs. *S. pinnata* L. grows naturally in Slovakia and is protected in this territory [1,2]. Traditional Chinese Medicine uses a decoction prepared from fruit of *Staphylea* L. as a cough remedy. The fresh roots are considered to have a blood-refreshing effect after delivery. The dried fruit is also used as a folk anti-diarrheal medicine [3]. Native Americans used the infusion from *S. trifolia* L. for its antirheumatic, dermatological, sedative and gynaecological activities. The seeds were considered sacred and were used in gourd rattles for dream and medicine dances [4,5].

To date, several compounds have been isolated from the leaves of *Staphylea* species. Previously we have described in details the main classes of chemical components and their amounts in various plant parts [6]. The significant antibacterial (against *Pseudomonas aeruginosa*, *Staphylococcus aureus* and *Enterococcus faecalis*) and cytotoxic activity of ethanol extracts from *S. holocarpa* and *S. pinnata* have been reported [7,8]. Significant antioxidant and immunomodulating activity have also been found in various polar (including water) and nonpolar *Staphylea* L. extracts [9,10].

The aim of our study was to investigate *in vitro*: 1) the biological properties, especially antiproliferative, cytotoxic and antioxidant activities, of aqueous leaf infusions from four *Staphylea* species: *S. colchica* (SC), *S. elegans* (SE) *S. holocarpa* (SH) and *S. pinnata* (SP) in human skin carcinoma cells (A431); 2) to compare the activities of these four species and 3), to find the compound or a group of compounds constituting the biological active component(s) in the extracts.

## 2. Results and Discussion

**Figure 1:**
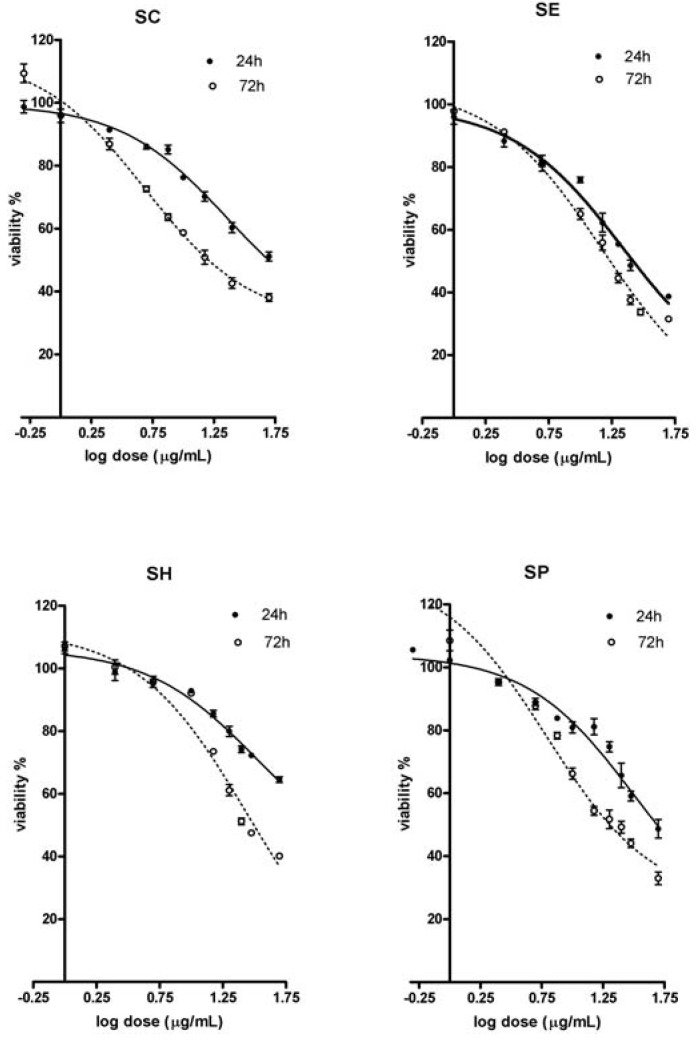
Antiproliferative activities (MTT test) of leaves water extracts from *Staphylea* L. The dose response curves after 24 and 72 h exposure of A431 skin carcinoma cells to tested extracts (SC - *S.colchica*; SE - *S. elegans*; SH - *S. holocarpa*; SP - *S. pinnata*).

As shown in Figure 1, proliferation of A431 human skin carcinoma cells treated with various doses of the tested extracts was inhibited in a time- and dose-dependent manner (MTT test). After a short treatment (24 h), the SE extract was the most effective one, and the the amount of live cells was significantly lower than seen with the SH and SC extracts. Extending the time of exposure increased antiproliferative activity of all extracts as proven by the decline n the number of surviving cells. After longer (72 h) exposure, the highest effect was induced by the SP (only ~31% of live cells), followed by the SE extract (~33.7%) and again, the less effective were the SC and SH extracts (37.2% and 40.2%, respectively). The shape of the dose response curves indicates a significant inhibition of cell growth in a generally narrow dosage range, the placement of the curves indicates that longer time of exposure induced higher cell sensitivity. ED_50_ values (e.g. the dose inducing 50% effect) expressing cell sensitivity are shown in Table 1. After 24 h SE and SC extracts were the most effective ones (ED_50_ for both were nearly identical). After 72 h exposure the cells demonstrated the highest sensitivity to SC extract the second most effective was SE extract. Antiproliferative activity of SH extract was the less efficient and was independent of exposure time.

**Table 1:** ED_50_ and maximal effects for MTT and LDH toxicity tests.

Species	Assay	Parameter	24 h	72 h
SC	MTT	ED50 (μg/mL) ± SE	20.1 ± 1.3	5.9 ± 0.9***
		Cell survival (%)	60.9 ± 11.1	37.2 ± 1.2
	LDH	ED50 (μg/mL) ± SE	32.4 ± 1.6	23.3 ± 2.4**
		max. effect (%)	32.1 ± 4.9	12.9 ± 2.3*
SE	MTT	ED50 (μg/mL) ± SE	18.3 ± 1.5	10.8 ± 1.9*
		Cell survival (%)	43.6 ± 2.8	33.7 ± 1.2*
	LDH	ED50 (μg/mL) ± SE	19.6 ± 1.3	13.7 ± 2.1*
		max. effect (%)	24.4 ± 1.7	10.5 ± 3.4*
SH	MTT	ED50 (μg/mL) ± SE	38.9 ± 3.0	16.3 ± 1.1***
		Cell survival (%)	66.6 ± 2.3	40.2 ± 0.6***
	LDH	ED50 (μg/mL) ± SE	38.3 ± 2.3	29.5 ± 2.0*
		max. effect (%)	32.5 ± 4.1	14.4 ± 0.9**
SP	MTT	ED50 (μg/mL) ± SE	35.3 ± 3.8	12.6 ± 1.9***
		Cell survival (%)	50.4 ± 3.8	30.9 ± 2.5*
	LDH	ED50 (μg/mL) ± SE	23.0 ± 1.3	17.5 ± 1.6*
		max. effect (%)	20.1 ± 1.8	21.2 ± 0.7

The values are mean ± SE, n=4, *p < 0.05; **p < 0.01; ***p < 0.001 (24 h vs. 72 h). (SC - S. *colchica*; SE - *S. elegans*; SH - *S. holocarpa*; SP - *S. pinnata*).

The highest accumulation of LDH in media was observed after treatment of A431 cells with SH and SC extracts (24 h) and SP (72 h). The less harmful effect was observed after treatment with SP (24 h) and SE (72 h) extracts. The decline of the LDH accumulation at 72 h interval (vs. 24 h) is only an apparent one. This phenomenon is due to: (a) daily replacement of growth media (with accumulated LDH) and (b) diminution of the live cells count. This explanation corresponds to reduced (~55–60%) LDH accumulation in supernatant and the presence of dead cells in the system (60 – 70%) after 72 h.

The data of MTT proliferation and LDH cytotoxicity tests proved SH extract as the less active one. Even the ED_50 _values (μg/mL) are similar for both, intracellular and plasma membrane injury after 24 h (MTT: 38.9 ± 3.0 vs. LDH: 38.3±2.3). Almost all data obtained confirm the SE extract as the most effective one. Its harmful effects are characterized by nearly identical ED_50_ values (μg/mL) regardless the time of exposure and test applied: 24h: 18.3 ± 1.5 (MTT) vs.:19.6 ± 1.3 (LDH); 72h: 10.8 ± 1.9 (MTT) vs. 13.7 ± 2.1 (LDH).

The antioxidant potential properties of tested extracts measured as DPPH radical scavenging activity (Table 2A) revealed that the most active extract was the one prepared from *S. elegans* (SE). The activity of individual extracts was in the following order, with significant differences between each other: *S. elegans* > *S. holocarpa* > *S. pinnata* > *S. colchica* .

To obtain more detailed information of the antioxidant activity in the above species and their extracts, the protective activity against peroxynitrite-induced tyrosine nitration has been investigated. This method did not disclosed any significant differences between individual extracts (Table 2B).

**Table 2:** Antioxidant activity of leaves water extracts.

Sample	DPPH radical scavenging activity [TE mmol/100 g](A)	Peroxynitrite scavenging activity [TE mmol/100 g](B)
SC	5.3 ± 0.3^a^	5.7 ± 0.4
SE	20.5 ± 0.3^b^	6.0 ± 0.4
SH	11.5 ± 0.1^c^	6.3 ± 0.3
SP	7.3 ± 0.3^d^	5.3 ± 0.1

The values are mean ± SE, n = 3; **A** – p < 0.001 for differences between values with different alphabet; **B** – NS; SC – *S. colchica*; SE – *S. elegans*; SH – *S. holocarpa*; SP – *S. pinnata*.

**Table 3:** Total content of polyphenols, flavons and flavonols in leaves water extracts.

Sample	Total content of polyphenols [GAE g/100 g] (A)	Total content of flavones and flavonols [QE g/100 g] (B)
SC	2.62 ± 0.04^a^	0.296 ± 0.008^+++^
SE	5.80 ± 0.05^b^	0.496 ± 0.003***
SH	4.81 ± 0.12^c^	0.414 ± 0.007**
SP	3.31 ± 0.04^d^	0.464 ± 0.004*

The values are mean ± SE, n = 3; Statistical significance for: **A** - values with different alphabet are statistically significant, p < 0.001; **B** –^+++^ p < 0.001 (vs SE, SH, SP), *** p < 0.001 (SE vs SH), ** p < 0.01(SP vs SH), * p < 0.05 (SE vs SP); *SC – S. colchica; SE – S. elegans; SH – S. holocarpa; SP – S. pinnata.*

**Figure 2:**
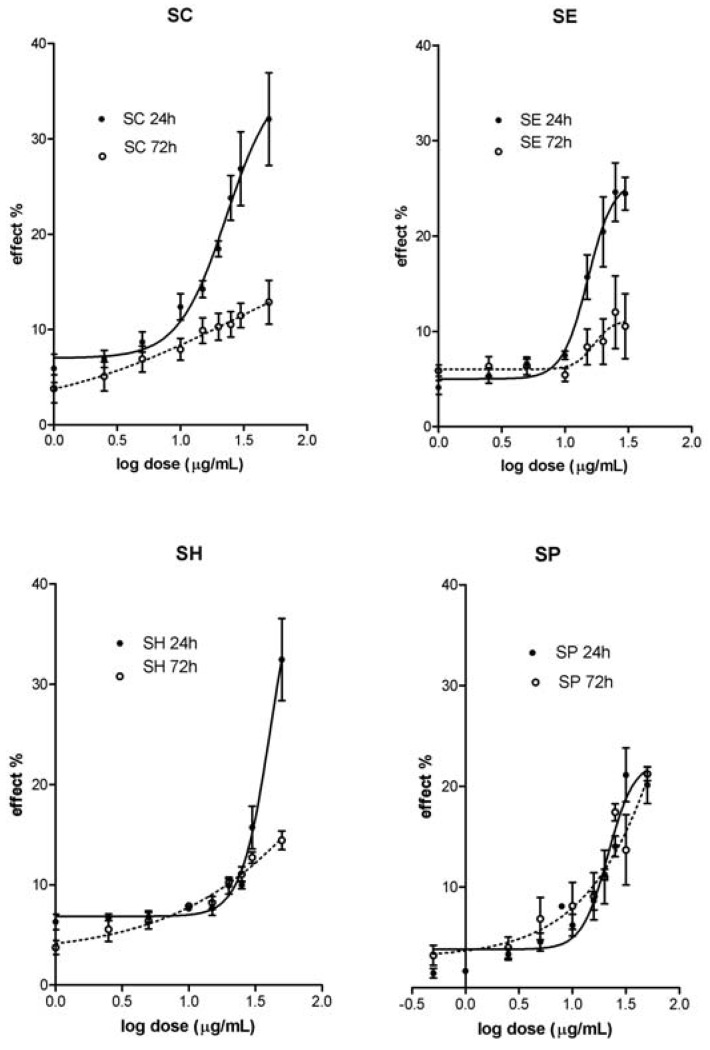
Cytotoxic activities (LDH leakage test) of leaves water extracts from *Staphylea* L. The dose response curves after 24 and 72 h exposure of A431 skin carcinoma cells to tested extracts (SC - *S.colchica*; SE - *S. elegans*; SH - *S. holocarpa*; SP - *S. pinnata*).

Table 3 shows significantly different contents of total polyphenols (3A) and flavones/flavonols (3B) in water extracts of individual *Staphylea* species. For both groups of biologically active compounds the lowest content was present in SC and the highest one in SE extract. The contribution of the above compounds to the observed biological activity was evaluated by regression analyses. The data did not confirm any relation between polyphenol content and either antiproliferative effect and membrane toxicity. The results support the association of flavonoids with cell death (MTT test: r = -0,8851, p < 0.05) and relation to cell membrane injury (LDH test: r = -0.8905, p < 0.05).

The highest content of polyphenols and flavonoids (Table 3) of the SE extract is accompanied by the highest antioxidant properties (Table 2A). Regression analyses proved that the DPPH antioxidant activity is only directly proportional to polyphenols content (r = 0.9565, p < 0.01), the relation to flavonoids content is at the statistical significance limit (r = 0.7119). 

Several compounds which have been isolated from the leaves of *Staphylea* species could be responsible for the observed activities. A large number of these substances are biologically active: flavonoids (rutin, quercetin, kaempferol, astragalin, 2-methyl-5,7-dihydroxychromon-7-*O*-β-D-gluco-pyranoside, nicotiflorine, kaempferol-3-neohesperidoside, kaempferol-3-*O*-[α-rhamnopyranosyl-(1→4)-rhamnopyranosyl-(1→6)-β-D-glucopyranoside, isoquercitrin) [11], proanthocyanidines (cyanidine), phenolic acids (caffeic acid, *p*-coumaric acid) [12], megastigmanes (icariside B_2_, 3*S*,5*R*, 6*R*,9*S*,7*E*-megastigman-7-ene-3,5,6,9-tetrol-9-*O*-β-D-glucopyranoside, staphylionosides A-K) [13], triterpenes and steroids (ursolic acid, betulinic acid, β-sitosterol and four other sterols), fatty acids (linolenic acid), cycloartenol, four tocopherols [14,15], pinnatanine, oxypinnatanine [16], noreugenine-7-*O*-glucoside [17].

## 3. Experimental

### 3.1. Plant material and extraction

The leaves of four *Staphylea* L. species were separately collected at the Dendrobiology Institute, Slovak Academy of Sciences - Arboretum Mlynany in May 2006 and authenticated by Ing. P. Hotka. The fresh leaves were dried at room temperature (22 °C) for 3 weeks.The dried leaves (10 g) were processed at a laboratory mill (Fritsch, Germany) and the water infusions were prepared according to the Czecho-Slovak Pharmacopoeia 4. (PhBs IV, 1987) The infusions were lyophilized and weighed. The yields were 10.1%; 11.0%; 10.6 % and 11.5% for SC; SE; SH and SP, respectively.

### 3.2. Cell culture

The use of human skin carcinoma cells was based on the historical external/local application of *Staphylea* water infusions in folk medicine [4,5]. A431 cells (a generous donation from INSERM U338, Strasbourg, France) were grown and passaged routinely as monolayer cultures in 75 mm^2^ flasks (Sarstedt, Germany). The cells were used at passage 10-20. The cells were seeded into 96-well plates at the density of 2 × 10^4^/well in D-MEM medium (Dulbecco`s modified Eagle`s medium) supplemented with 10% FBS (fetal bovine serum, Gibco BRL, Invitrogen, Paisley, Scotland), antibiotics (penicillin 50,000 U/L, streptomycin 50 mg/L, Cambrex, USA) and cultured in a humidified atmosphere of 5% CO_2_ at 37 °C. The cells were treated with various doses (1–150 µg/mL) of SC, SE, SH and SP water extracts for 24 and 72 h. The culture medium and the tested extracts were refreshed every 24 h. This experimental design was preferred as to avoid possible undesirable effect of metabolites, to supply surviving cells with nutrients and to measure repeated dosage effects. Control cells were incubated in culture medium only.

### 3.3. MTT cell proliferation assay and LDH cytotoxicity assay

MTT viability assay was employed to assess cell growth. The proliferation test is based on the color reaction of mitochondrial dehydrogenases from living cells with MTT (3-[4,5-dimethylthiazol-2-yl]-2,5-diphenyltetrazolium bromide, Sigma, USA). At the end of the treatment period, MTT (final concentration 1 mg/mL) was added to each well, which was then incubated at 37 ^o^C in 5% CO_2_ for four h. The colored crystals of produced formazan were dissolved in DMSO (dimethyl sulfoxide, Sigma, USA). The absorbance at 630 nm was measured using EL× 800 Microplate Reader (Bio-Tek Instruments). The effect of *Staphylea* water extracts on the cell proliferation was calculated as the effect (%) of individual extract dose vs control, untreated cells. The LDH cytotoxicity assay measures cell membrane integrity. The assay detects the release of stable cytosolic enzyme LDH into the culture medium, due to cell membrane injury. The activity of the enzyme was measured using CytoTox 96^®^ Non-Radioactive Cytotoxicity Assay Kit, (Promega, USA) in accordance with the manufacturer’s instructions. At each time point, the data were normalized to the amount of LDH spontaneously released from control, untreated cells. The effect was expressed as the ratio (%) of released LDH (LDH in media) / total LDH content (in media + LDH activity in cells). The highest dose 50 µg/mL of each extract was used for calculating cell survival (MTT test) and max. effect (LDH test).

### 3.4. DPPH radical reduction

Reduction of DPPH radicals was measured by a Synergy HT multiplate-reader (BIO-TEK). The test solution in DMSO (50 μL, 1 mg/mL) was mixed in a 96-well microplate with 0.25 mM MeOH solution of DPPH (200 µL). After 5 min, the absorption was measured at 517 nm. Quantification was calculated from the Trolox^®^ calibration curve. The results were expressed as Trolox^®^ equivalent antioxidant capacity (TEAC) per 100 g of dry weight of leaves extract.

### 3.5. Peroxynitrite scavenging activity

The HPLC method for measuring the inhibition of peroxynitrite mediated tyrosine nitration was used. The method consists of mixing peroxynitrite solution (8 μL, 10 mM) in 0.1 M NaOH with 1.0 mM solution of tyrosine in 0.05 M phosphate buffer pH 6.0 (42 μL) containing the sample (0.5 mg/mL) and DMSO (in a 1:1 ratio with water) in the HPLC injector. The reaction mixture was injected directly in the HPLC system (HP1100 with autosampler, quaternary pump and diode-array detector, Agilent Technologies, Germany). The separation was carried out with a Supelcosil ABZ+Plus column (25 cm × 4.6 mm, 5 μm; Supelco, USA); mobile phase consisted of 90% 40 mM HCOOH and a 10% CH_3_CN (v/v) at a flow rate 1 mL/min. The chromatograms were detected at 356 nm. Inhibition of tyrosine nitration was calculated relative to peak area of 3-nitrotyrosine founded in the control measurement. The percentage of inhibition of tyrosine nitration was compared to that of the calibrated Trolox^®^ standard. Results were expressed as Trolox^®^ equivalent antioxidant capacity (TEAC) per 100 g of dry leaves extract weight.

### 3.6. Determination of flavones and flavonols content

Flavones and flavonols contents were analyzed by a colorimetric method. The test solution in DMSO (150 μL, 1 mg/mL), 1.3% aluminium chloride (in MeOH, 25 μL) and DMSO (100 μL) were mixed in a 96-well microplate. After 30 min, the absorption was measured at 425 nm in Synergy HT microplate-reader (BIO-TEK). Each measured absorbance was reduced by subtraction of a sample blank value (without aluminium chloride). The calibration curve was plotted versus concentrations of quercetin. The results were expressed as quercetin equivalent (QE) per 100 g of dry leaves extract weight.

### 3.7. Determination of phenolic compounds content

The total content of phenols was determined by a modified Folin-Ciocalteau colorimetric method. The test solution in DMSO (1.5 mL, 1 mg/mL) was transferred to a 10 mL volumetric flask, the Folin-Ciocalteau reagent (400 μL) was added and after 3 min, sodium carbonate (Na_2_CO_3_) solution (75 g/L) was added. After 2 hours, the suspension was centrifuged (5,000 r.p.m., 5 min) and the absorbance of the supernatant was measured at 760 nm. The calibration curve was plotted versus concentrations of gallic acid. The results were expressed as a gallic acid equivalent (GAE) per 100 g of dry leaves extract weight.

### 3.8. Statistical analysis

The data are expressed as the means ± standard error (SE). Biological activity is the result of four individual experiments performed in duplicates for each dose. The effects of each extract were expressed by ED_50_ (*e.i.,* the dose required for a 50% response) and by the magnitude of maximal effect in exposed cells. The ED_50_ values were calculated from the dose response curves by a computer program (GraphPad Prism). Statistical differences between time intervals and individual extracts were evaluated by one-way ANOVA and Bonfererroni *post hoc* test.

## 4. Conclusions

The present study describes biological activities of four water *Staphylea* L. leaves extracts. The results indicate that water extract from *S. elegans* possess the highest antiproliferative, cytotoxic, as well as antioxidant activity and is characterized by considerable polyphenol and flavonoid content. *S. holocarpa* is the least toxic. The antiproliferative/cytotoxic effect is related to the presence of flavonoids and the antioxidant activity is associated with polyphenol content. The study demonstrates that water extracts from leaves of various *Staphylea* L. species differ, not only in the contents of biologically active compounds, but also by the extent of harmful and beneficial effects. It is possible that other secondary metabolites, like triterpenes and steroids [18], may contribute to the observed biological activities.
